# A Survey of Deep Learning Road Extraction Algorithms Using High-Resolution Remote Sensing Images

**DOI:** 10.3390/s24051708

**Published:** 2024-03-06

**Authors:** Shaoyi Mo, Yufeng Shi, Qi Yuan, Mingyue Li

**Affiliations:** 1 College of Civil Engineering, Nanjing Forestry University, Nanjing 210047, China; moshaoyi@njfu.edu.cn (S.M.); yq@njfu.edu.cn (Q.Y.); 2 School of Foreign Studies, Nanjing Forestry University, Nanjing 210047, China; mylee@njfu.edu.cn

**Keywords:** road extraction, high-resolution remote sensing images, deep learning, supervised learning, network model

## Abstract

Roads are the fundamental elements of transportation, connecting cities and rural areas, as well as people’s lives and work. They play a significant role in various areas such as map updates, economic development, tourism, and disaster management. The automatic extraction of road features from high-resolution remote sensing images has always been a hot and challenging topic in the field of remote sensing, and deep learning network models are widely used to extract roads from remote sensing images in recent years. In light of this, this paper systematically reviews and summarizes the deep-learning-based techniques for automatic road extraction from high-resolution remote sensing images. It reviews the application of deep learning network models in road extraction tasks and classifies these models into fully supervised learning, semi-supervised learning, and weakly supervised learning based on their use of labels. Finally, a summary and outlook of the current development of deep learning techniques in road extraction are provided.

## 1. Introduction

There are various types of roads in remote sensing images, such as urban roads, suburban roads, mountain roads, expressways, overpasses, etc. As the resolution of remote sensing images continues to improve, high-resolution images contain more information about the texture, shape, structure, and neighborhood relationships of roads compared to low- and medium-resolution remote sensing images, enabling more accurate road information extraction [1]. Extracting road information from high-quality remote sensing images has always been challenging due to multiple factors. These include complex and cluttered backgrounds (such as buildings, vegetation, and various road types), diverse road shapes (which vary in width and length), and poor image perspectives (resulting from occlusions by clouds and fog, as well as lighting effects). Furthermore, as urban areas expand, the topological structure of roads becomes exceptionally complex, with numerous buildings obstructing large portions of road areas [2].

Road extraction is typically regarded as a semantic segmentation task, where road and non-road labels are assigned to all pixels in an image, achieving binary semantic segmentation. With the rapid advancement of deep learning, there has been widespread interest in its powerful data fitting and information processing capabilities. Previous reviews have focused on the progress of road extraction techniques in remote sensing images. They summarize both traditional and deep learning methods. For instance, Abdollahi et al. [3] summarized road extraction methods in remote sensing imagery as being based on deep learning techniques, such as DCNN [4], FCN [5], deconvolution [6], and GANs [7]. Lian et al. [8] further categorized extraction methods into heuristic and data-driven road extraction approaches. Heuristic methods predominantly employ semi-automatic or fully automatic traditional techniques for road extraction, such as snake model-based contour extraction [9], geodesic path-based approaches [10], dynamic programming-based methods [11], and template matching [12]. Automated extraction methods include machine learning segmentation algorithms like SVM [13], K-Means [14], and Bayesian classifiers [15], edge analysis-based methods [16], and map-based techniques [17]. The data-driven module, based on [3], also adds a summary of graph-based methods [18]. Jia et al. [19] discussed the applications of active and passive remote sensing technologies in road extraction, including high-resolution, hyperspectral, synthetic aperture radar (SAR), and airborne laser scanning (ALS) technologies, and also provided a summary of the current state and future prospects of multi-source data fusion. Liu et al. [20] summarized previous data-driven methods as fully supervised learning methods and introduced weakly supervised and unsupervised learning methods. Currently, mainstream road extraction network models can be broadly categorized into fully supervised and semi-supervised (weakly supervised) extraction. The differentiation between these two learning methods primarily depends on whether the model requires substantial label data support during training. Fully supervised learning relies on a large number of pixel-level training labels for model training. This approach often achieves high-precision segmentation structures, but its generalization capability is relatively weak, resulting in limited segmentation performance in unknown scenarios. Moreover, obtaining pixel-level labels often requires a significant amount of manual annotation work, and these annotated data exhibit a high degree of subjectivity, potentially impacting the accuracy of road segmentation by the model. Semi-supervised (weak) learning relies on fewer training label data, which can be in the form of points, lines, and other weak labels for model training. While semi-supervised (weak) learning generally lags behind in segmentation performance compared to fully supervised learning, it offers certain advantages. This approach reduces the dependency on label data, thus alleviating the burden of manual annotation.

**Figure 1:**
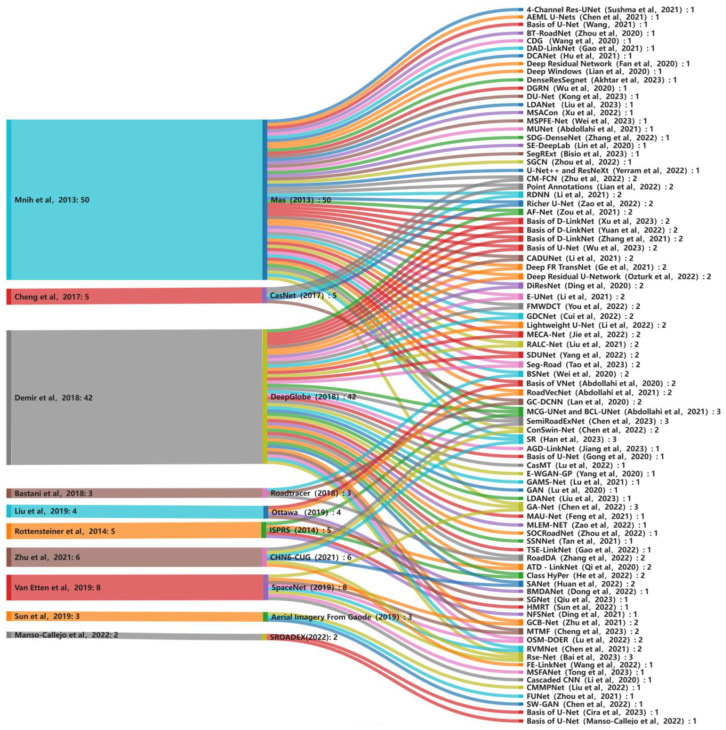
Public datasets used more than twice from 2020–2023.

To address issues of insufficient labels and high annotation costs in road extraction tasks https://www.isprs.org/education/benchmarks/UrbanSemLab/ (accessed on 2 March 2024), this paper classifies network models based on the use of pixel-level labels, including fully supervised learning, semi-supervised learning, and weakly supervised learning. In this paper, “road extraction”, “deep learning”, and “remote sensing” were chosen as searching keywords. The Web of Science (WOS) and Google Scholar databases were used as literature search tools to primarily retrieve relevant literature from 2020 to 2023. We organized the publicly available datasets mentioned in the retrieved literature over 40 datasets (2013–2023). This compilation includes 22 publicly accessible road datasets, with images primarily sourced from Google Earth, OpenStreetMap (OSM), open APIs, drone imagery, and satellite imagery, covering urban, suburban, rural, and forested areas. Furthermore, we observed that multiple publicly available road datasets such as Massachusetts [21], ISPRS^1^, CasNet [22], DeepGlobe [23], SpaceNet [24], Roadtracer [25], Ottawa [26], and CHN6-CUG [27] were utilized two or more times between 2020 and 2023, as depicted in Figure 1. In Figure 1, the leftmost column represents the number of times datasets were used during these four years, while the rightmost column indicates the number of times corresponding network models utilized the datasets. Additionally, we conducted research on pre-processing and post-processing work related to remote sensing images in the relevant literature. For instance, a real-time multi-temporal color data enhancement technique was introduced for improving Sentinel-1 multi-polarization and Sentinel-2 multi-spectral imagery datasets [28]. Image quality was enhanced through the application of the contrast-limited adaptive histogram equalization (CLAHE) algorithm to mitigate mountain shadow issues [29]. Post-processing tasks included road vectorization [30], road information, and label reconstruction [31], among others. Due to space constraints, this paper primarily focuses on the analysis and discussion of road feature extraction research based on fully supervised deep learning network models. The structure of this paper is as follows: Section 1 introduces and briefly elucidates the challenges and methods in the field of road extraction from remote sensing images. Section 2 delves into road feature extraction using fully supervised deep learning network models while studying the strengths and limitations of these network models. Section 3 explores road feature extraction through semi-supervised (weak) deep learning. Section 4 presents a comprehensive review of road extraction methodologies, conducting a comparative analysis of diverse models in terms of their performance. Ultimately, we objectively discuss the limitations inherent in current supervised learning models. Section 5 put forwards future prospects of road extraction and challenges.

## 2. Road Feature Extraction Based on Fully Supervised Deep Learning Network Models

**Figure 2:**
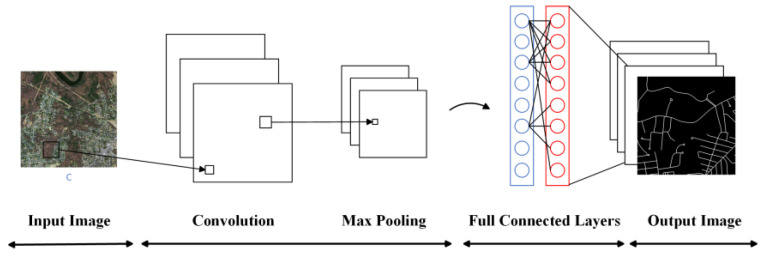
Patch-based CNN model.

**Figure 3:**
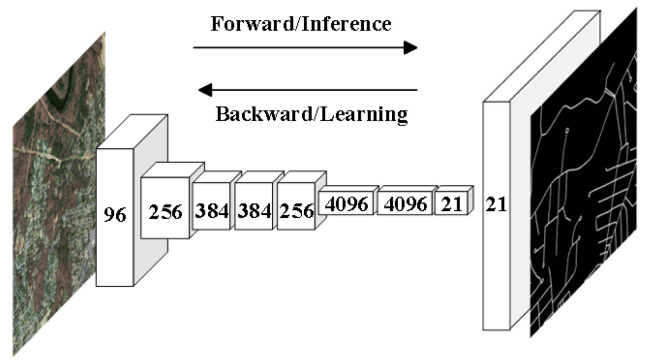
Fully Convolutional Neural Network (FCN) model.

Mnih [32] first introduced convolutional neural networks (CNNs) into road extraction tasks. Initially, in the field of deep learning for road extraction, many researchers used block-based CNN models to process roads within images. For example, finite state machine (FSM) and patch-based CNN (as shown in Figure 2) methods were employed [33] to track and extract roads separately. These patch-based CNN models performed excellently in aerial images with a spatial resolution of 1.2 m but struggled to achieve satisfactory results in higher-resolution (0.15 m) image extraction. To address this issue, Rezaee and Zhang [34] improved traditional patch-based CNN methods, enabling them to outperform support vector machine (SVM) methods in road extraction from high-resolution image datasets (0.15 m spatial resolution). However, patch-based CNN methods overly relied on the sliding window approach, which involved feature extraction through convolutional and pooling layers, followed by backpropagation to fine-tune the final parameters. This resulted in relatively low extraction efficiency, which was insufficient for meeting the requirements of practical applications. Additionally, choosing an appropriate sliding window size was a challenging task. It was not until the emergence of fully convolutional neural networks (FCNs), that this problem was effectively solved. The FCN model was first introduced into the field of image segmentation [35], as shown in Figure 3, and it significantly improved segmentation efficiency. In contrast to traditional patch-based CNN models, an FCN is capable of pixel-level image classification, meaning it classifies each pixel into a category, with the output providing the category for each pixel. The FCN replaces fully connected layers with convolutional layers, achieving end-to-end semantic segmentation. This overcomes the inefficiency issue of patch-based CNN methods and allows for the extraction of target semantic information while preserving spatial information [1]. While the FCN enhanced the CNN by enabling pixel-to-pixel classification, it disregarded the relationships between pixels. Therefore, subsequent models introduced various attention mechanism modules to strengthen the relationships between pixels. Furthermore, the FCN’s structure has offered novel insights into encoder–decoder network architectures.

### 2.1. Road Feature Extraction Based on Encoder–Decoder Structure

**Figure 4:**
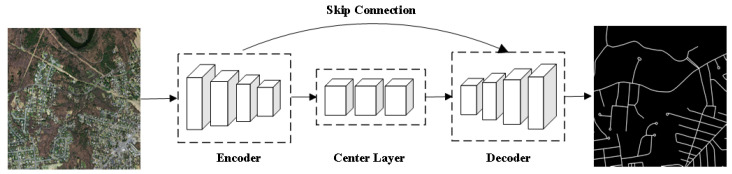
Network Models Based on Encoder–Decoder Structures.

Following the FCN, network structures based on encoders and decoders have emerged and been widely applied. Their operation involves multiple downsampling of the original image by the encoder to obtain multi-level image feature information, followed by upsampling through the decoder to restore spatial information (Figure 4). Models based on this structure include SegNet [36], U-Net [37], PSPNet [38], LinkNet [39], DeepLab V3+ [40], and more. Among them, U-Net is one of the most classic networks with a symmetrical U-shaped encoder–decoder structure, initially applied in medical image segmentation tasks. This model employs an encoder–decoder structure for multi-scale feature fusion and pixel-level classification, while utilizing skip connections to acquire spatial information from the encoder and achieve feature fusion. The U-Net was extended by Chen et al. [41] to propose the Reconstruction Bias U-Net network. They added the ReLU function and a maxpooling layer and introduced decoding branches in the decoder to capture multiple semantic information from various upsampling processes. At present, there is a profusion of road extraction models based on encoder–decoder structures, encompassing models like LinkNet, D-LinkNet [42], U-Net and its variants VNet [43], U-Net++ [44], U^2^-Net [45], Dense-UNet [46], Res-UNet [47], MC-UNet [48], and others. While their structures exhibit slight variations, the primary distinctions lie in the encoder and decoder backbone models, intermediate layers, skip connection layers, and network model optimizations. In recent years, the rapid development of transfer learning has facilitated model training, especially when dealing with limited training data, significantly reducing training time and costs. Many scholars use network models pre-trained on ImageNet, such as VGG [49] and ResNet [50], as the backbone structure for their models. For instance, the pre-trained VGG16 from ImageNet was introduced by DeepLab V1 [51], along with the proposal of spatial convolution (dilated/atrous convolution) to increase the receptive field, addressing the issue of reduced resolution due to repeated pooling and downsampling. ResNet-50 was adopted as the backbone structure for PSPNet, which introduced spatial pyramid pooling (SPP) to gather contextual information from different regions, thereby enhancing its ability to obtain global information. DeepLab V2 [52] replaced the VGG16 backbone of DeepLab V1 with ResNet-101 and, inspired by SPP, introduced atrous spatial pyramid pooling (ASPP) to integrate multi-scale information. The emergence of SPP and ASPP resolved the issue of needing to resize images before they enter the neural network, especially for fixed-size inputs like 224 × 224 images. At present, some scholars introduce SPP and ASPP modules into models to enhance the extraction of road features from images through feature fusion. Lan et al. [53] and Gao et al. [54] have respectively proposed the GC-DCNN and Tes-LinkNet models based on the U-Net and LinkNet models. The former introduces the SPP module into the intermediate layers, while the latter uses the ASPP module. Huan et al. [55] introduced the SANet model pre-trained with ResNet-50 and introduced the ASPP module in the encoder. Inspired by dense convolution, Q. Wu et al. [56] introduced the dense and global spatial pyramid pooling module (DGSPP) into the decoder and encoder to enhance the network’s perception and aggregation of contextual information. Wei and Zhang [57] integrated the multi-level strip pooling module (MSPM) into the skip connection layers to ensure road connectivity by aggregating long-range dependencies from different levels. LinkNet used ResNet-18 as the encoder backbone and improved segmentation efficiency by directly connecting the encoder and decoder. D-LinkNet employed the pre-trained ResNet-34 as the encoder backbone and introduced dilated convolutions in the intermediate layers. The design of D-LinkNet includes four progressively larger dilated convolution layers, forming a stacked pyramid pattern, also known as the D-Block, making the output of each layer the input to the next. This design expands the receptive field while maintaining image resolution, contributing to its championship in the DeepGlobe 2018 Road Extraction Challenge. However, there is a potential issue with the dilated convolutions in the intermediate layers of the D-LinkNet model, as it may lead to the loss of continuous information between neighboring pixels and introduce some unrelated contextual information, affecting road extraction’s connectivity and integrity. Therefore, some scholars have enhanced the dilated convolutions in the intermediate layers of the D-LinkNet model. Gong et al. [58] replaced dilated convolutions with dense dilated convolutions, enabling multi-scale information fusion while expanding the receptive field. Wang et al. [59] restructured the D-Block into the DP-Block, inspired by the pyramid attention network [60]. They introduced global pooling and designed dense connections between convolutions to fully utilize global and dense information for enhancing road features. J. Zhang et al. [61], on the other hand, took inspiration from MobileNet V2 [62] and introduced bottleneck modules (bottleneck block) within the D-Block, forming D-Blockplus, thereby reducing network parameters and improving network performance.

### 2.2. Road Feature Extraction Based on Feature Fusion

Feature fusion refers to the combination and superimposition of features from different layers or branches using techniques such as weighting or concatenation. These features possess distinct characteristics. Low-level features have higher resolution, containing more positional and detailed information, but due to fewer convolutions, their semantic information is relatively less and may contain some level of noise. High-level features, on the other hand, contain richer semantic information but have lower resolution and a less effective ability to perceive detailed information. Feature fusion employs various strategies, such as feature concatenation, feature summation (including mean, pooling, weighted summation, like ASPP and SPP mentioned earlier), element-wise multiplication of feature elements, skip connections, deconvolution, attention mechanisms, and multi-scale feature fusion. These methods comprehensively utilize features of different levels and properties, making them a crucial component in network models.

#### 2.2.1. Feature Fusion Based on Attention Mechanisms

The attention mechanism is a crucial module in deep learning networks and is considered as an additional neural network that can effectively integrate with neural networks [63]. In road feature extraction research, issues such as fragmented extraction results and poor connectivity often arise due to obstructions from buildings, trees, or background interference with similar textures. In such cases, by appropriately introducing attention modules, the model can focus more on information at road edges and intersections, leading to more connected and complete road extraction results.

In recent years, attention mechanisms have gained considerable traction in the domain of road extraction. Extensive research has delved into self-attention, channel attention [64], spatial attention [65,66], and hybrid attention mechanisms [67]. The integration of the multi-head attention mechanism from Transformer [68] into architectures like ConSwin-Net [69] and Seg-Road [70] has effectively addressed the limitations of conventional CNNs, markedly enhancing the ability to perceive road texture intricacies and contextual information. Modules like the self-attention feature transfer module (SAFM) [71] have further facilitated comprehensive information integration within models, significantly bolstering the performance and robustness of road extraction tasks.

The foundational mechanisms of the channel attention module (CAM) and spatial attention module (SAM) play pivotal roles in road extraction. Networks such as Nested SE-DeepLab [72] and RALC-Net [1] have overcome challenges in road feature extraction by leveraging the squeeze-and-excitation (SE) and residual attention (RA) modules. Additionally, the incorporation of serial or parallel attention mechanisms like the convolutional block attention module (CBAM) [73] and ProCBAM [74] markedly improved the network’s focus on road information, thereby elevating the performance of road extraction tasks. These innovative methods and varied applications of attention mechanisms comprehensively showcase effective strategies for enhancing model performance in road extraction tasks, enabling more efficient capture of road-related information. We have summarized the prevalent attention mechanism modules in current road extraction tasks in Table 1.

#### 2.2.2. Feature Fusion Based on Multi-Scale Images

The term “multi-scale” refers to images of different resolutions or different levels of image features (low-level features, high-level features). The purpose of feature fusion is to explore how to effectively utilize these multi-scale images to obtain more accurate road feature information [85].

The design of multi-scale feature fusion modules often draws inspiration from parallel or serial multi-branch network architectures, such as feature pyramid networks (FPNs) [86], Inception [87], and HRNet [88]. This section provides an overview of the multi-scale feature fusion modules and methods employed in road image segmentation tasks. Researchers have utilized supervised learning by combining edge information with image features to enhance road image segmentation networks. Various module designs have been proposed to address issues related to extracting road shapes and enhancing connectivity, such as the multi-scale context augmentation module [89], spatial context module [90], and feature review module [91]. Some modules are particularly adept at capturing elongated road shapes, while others focus on enhancing global features. Additional modules aim for multi-scale feature fusion. Solutions tailored for narrow, continuous, and expansive roads in high-resolution remote sensing images have also been proposed, incorporating multiple modules to optimize spatial feature preservation, shape enhancement, and multi-feature fusion. These innovative modules and methods collectively drive advancements in road extraction tasks, providing crucial technical support for more accurate identification of road shapes and improved segmentation outcomes. Due to space limitations, detailed method characteristics are summarized in Table 2.

#### 2.2.3. Feature Fusion Based on Multi-Modal Fusion

Solely relying on optical remote sensing imagery to provide learning information for network models does not guarantee excellent learning outcomes. This is due to spectral similarities between buildings and roads and the potential for occlusions caused by tall buildings and trees. These factors can lead to inaccurate identification and acquisition of road feature information by the model, ultimately affecting road extraction results. Additionally, sensor imaging and lighting conditions can also adversely affect the recognition and acquisition of road feature information. Recognizing this challenge, researchers have explored multi-modal data, including multi-spectral (hyperspectral) data, synthetic aperture radar (SAR) [99], light detection and ranging (LiDAR), unmanned aerial vehicle (UAV) data, GPS trajectory data, and multi-temporal data. The penetrative and oblique observation properties of synthetic aperture radar (SAR) have been ingeniously leveraged by J. Zhang et al. [61] to address issues arising from shadows and occlusions caused by vegetation and buildings in optical remote sensing, providing network models with more detailed road information. On the other hand, dual-temporal optical remote sensing imagery has been employed [100] to detect and update road databases. Sensors with high revisit times, such as Sentinel-1 and Sentinel-2, have been utilized by Ayala et al. [28] to enhance datasets with multi-temporal multi-spectral and SAR data through color data augmentation.

Multi-modal fusion involves feature integration between different data sources, particularly for cross-source fusion between GPS trajectory data and remote sensing imagery. Similarly, we have provided a more intuitive tabular summary of methods related to multi-modal feature fusion in Table 3.

Attention mechanisms themselves are models with advantages such as fewer parameters, faster processing speed, and good performance. Compared to CNNs, attention mechanisms have lower model complexity, fewer parameters, and lower computational requirements. Furthermore, attention mechanisms address the issue of non-parallel computation in RNNs [105], as they do not rely on the results of the previous step, enabling efficient parallel computation. Hence, they have become an important component of feature fusion in network models. However, it is worth noting that the introduction of attention mechanisms may lead to model overfitting. If a network model is already complex, incorporating attention mechanisms can increase the number of model parameters, potentially causing overfitting issues. Additionally, fusing different features together may introduce noise and other challenges. Attention itself is a type of feature, so when integrating it with other features, careful consideration is needed to assess whether it might negatively impact the network model’s performance. For multi-modal data, while it provides richer semantic information to networks, there may be differences in semantics among different modalities. Therefore, addressing noise reduction and semantic differences while fusing these features is an issue to be focused on in the future.

### 2.3. Road Feature Extraction Based on GANs

**Figure 5:**
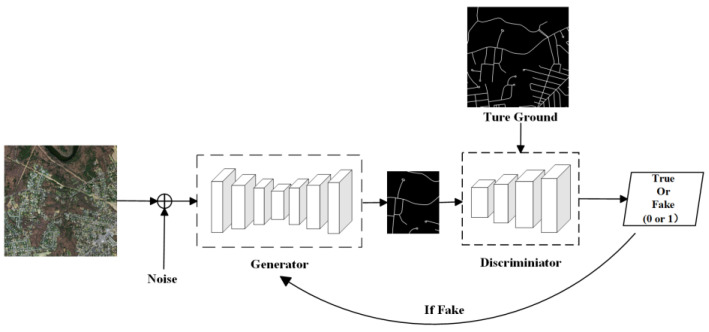
Network Model Based on Conditional Generative Adversarial.

In 2014, generative adversarial networks (GANs) were introduced by Goodfellow et al. [106] operating on an unsupervised learning approach, consisting of a generator G and a discriminator D. The task of the generator is to generate data closely resembling real images, attempting to “deceive” the discriminator. The discriminator’s role is to determine whether the data generated by the generator is correct and provide feedback to enhance the generator’s ability to “fabricate”. This process forms a cycle, continuing until neither can deceive the other. Essentially, it is a zero-sum game, also known as the Bash game. However, because the generator does not require training labels, data can be generated too freely, including images, text, or even sound from noise, which is not ideal for image recognition tasks. To address this issue, the introduction of some conditions to both the generator and discriminator was proposed. In the context of image recognition tasks, conditions could be introduced to the discriminator to make it generate only images. In the same year, conditional generative adversarial networks (CGANs) [107] were introduced (Figure 5). CGANs are generative adversarial network models with constraint conditions. Incorporating variables y into both the generator and discriminator, these variables guide the data generation process by the generator. The variables y can be labels or even images, marking a shift of GANs from unsupervised learning towards supervised learning.

In 2017, the Pix2pix [108] model was introduced, which is based on the structure of conditional generative adversarial networks (CGAN) for image-to-image transformations, also referred to as domain adaptation. In this approach, the generator of the model utilizes a U-Net network, while the discriminator is designed using the PatchGAN architecture. Many researchers continue to reference this model in current road extraction tasks. For instance, Yang and Wang [109] followed the structure of Pix2pix and introduced the WGAN-GP network for rural road extraction. They used both U-Net and BiSeNet as generators, employing an ensemble strategy to combine their inference outputs for better road vector generation. The discriminator in their model used PatchGAN. Cira et al. [110,111] applied the Pix2pix model to post-process road extraction. They improved the integrity of road surface area extraction by contaminating labels and reconstructing them. In addition, Abdollahi et al. [7] proposed a deep learning approach using conditional generative adversarial networks (CGANs) for road segmentation in high-resolution aerial imagery. They utilized an enhanced U-Net model (MUNet) as a generator to segment images and obtain high-resolution segmented maps of road networks. NIGAN [112], comprising two CGAN networks, was used for scene selection in mountainous road scenarios. This was caried out to pre-select areas that contain mountainous road scenes, thereby reducing the workload in subsequent segmentation and road extraction tasks. The generator in their model is based on an encoder–decoder structure, utilizing ResNet-34 as the backbone. Middle layers incorporate dilated convolutions, which are helpful for extracting small objects like roads and expanding the receptive field while enhancing global information.

Conditional generative adversarial networks (CGANs) have played a crucial role in road extraction tasks. They are not only used for road segmentation but also for pre-processing road extraction, enriching road information in images, and reducing the workload for subsequent segmentation networks. Additionally, in post-processing, employing adversarial training techniques to enhance segmentation results has reduced issues related to fragmentation while improving road connectivity.

### 2.4. Road Feature Extraction Based on Cumulative Integration of Multiple Models

**Figure 6:**
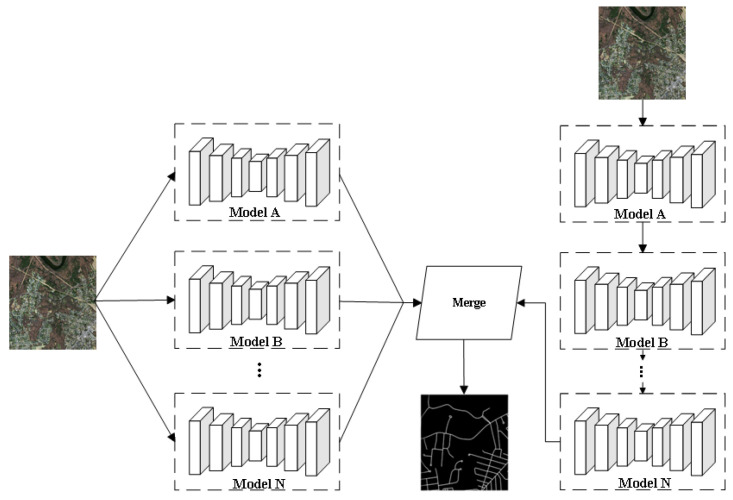
Network Model Based on Cumulative Integration of Multiple Models.

In road extraction tasks, ensemble strategies have been increasingly adopted by researchers to combine multiple models serially or in parallel. Integrated models with strong generalization capabilities, high robustness, and exceptional segmentation performance have been highly sought after in research endeavors. Parallel strategies (Figure 6) are most commonly used. For example, Senthilnath et al. [113] employed three relatively mature network models, FCN-32, Pix2Pix, and CycleGAN [114], for transfer learning. Both Pix2Pix and CycleGAN are commonly used in domain transfer tasks. The key difference is that Pix2Pix requires training data to be in pairs, which is challenging to find in the natural world. The emergence of CycleGAN effectively solves this problem. They proposed the Deep TEC integrated classifier, which utilizes a parallel strategy to integrate the results of road segmentation from three models. This approach achieved outstanding integration performance in extracting urban road networks from drones. Cira et al. [115] combined improved CNN, VGG, ResNet-50, and Inception-ResNet [116] models in parallel and fused extraction results using an averaging structure. This strategy aims to leverage the strengths of each model while minimizing their weaknesses, ultimately resulting in a classifier with reduced classification error. Chen et al. [117] employed ResNet-50 models with three distinct convolution kernel sizes for road extraction, integrating the results to form a ResNet-50 training block enriched with high-level information. Li et al. [118] reorganized the layers of U-Net and duplicated a single submodel N times, creating an ensemble model E consisting of N parallel submodels. Following optimization and prediction, they ultimately established an E-UNet model with 14 layers. Abdollahi et al. [119] adopted a parallel approach by linking two improved U-Net models, BCL-UNet (ConvLSTM [120] + U-Net) and MCG-UNet (BConvLSTM + SE + dense convolutions [121]). They introduced dense convolutions and compression activation modules in the upsampling layers of the standard U-Net. They employed bidirectional convolutional long short-term memory (BConvLSTM) for skip connections, enabling the generation of high-resolution segmentation maps even in challenging backgrounds while preserving edge information. The graph-based dual convolutional network (GDCNet) [122] integrates graph convolutional networks (GCNs) and CNNs. Employing a ResNet-50 backbone that included encoder and decoder convolutional neural networks, researchers applied a parallel approach for road extraction, effectively addressing concerns associated with poor connectivity and discontinuities. This was achieved by generating complementary spatial–spectral features at both superpixel and pixel levels and efficiently propagating these features between graph nodes and image pixels using a graph decoder. Sun et al. [123] employed a parallel network model consisting of dual branches for road and building extraction. One branch is the multi-resolution semantic extraction branch, composed of three parallel ResNet networks, used to extract semantic features of roads and buildings at different resolutions. The other branch is the Transformer semantic extraction branch, which utilizes a ResNet-18 backbone and features a Transformer-based encoder–decoder. This parallel strategy successfully addresses the current limitation of semantic segmentation networks in terms of receptive field by fusing the output results of the two branches.

Certainly, a serial strategy employing multiple models for road extraction is also utilized by some researchers. For instance, a direction-aware residual network, DiResNet [124]. DiResNet comprises a ResNet segmentation network (DiResSeg) based on the decoding layers with structural supervision and a refinement network (DiResRef) based on U-Net. The former is dedicated to enhancing the learning of road topology, while the latter further refines the road segmentation results. Z. Chen et al. [125] drew inspiration from the AdaBoost classification algorithm and combined multiple lightweight U-Net models by connecting them in a serial manner, forming AdaBoost-like end-to-end multiple lightweight U-Nets (AEML U-Nets). Under this serial strategy, the output of the previous network serves as the input for the next one. To ensure the training quality of each U-Net, the researchers designed a multi-objective optimization strategy for joint training of all U-Nets. Finally, the output results of each U-Net are fused to obtain the ultimate road extraction result.

With the continuous development of deep learning, models are gradually evolving towards greater depth and width. However, it is important to note that increasing depth and width does not always lead to improved model performance and can potentially result in issues like overfitting. In this section, we summarize how scholars leverage the unique characteristics of different models and employ ensemble strategies to integrate these models. These characteristics include having fewer model parameters, fast recognition speed, strong generalization, and expertise in extracting road features in various scenarios. By combining multiple models, whether they are simple or mature, researchers have achieved better road feature extraction results than with a single model. Nonetheless, it is essential to be aware that multiple independent models do not always outperform a deeper and larger single model. This is because these models are trained independently, and their training outcomes may vary. In parallel extraction, individual models may perform poorly, becoming bottlenecks for overall performance. In serial extraction, if the same model is used for serial processing, it may lead to a series of problems. For instance, determining strategies to ensure consistent training results for each model and whether an excessive number of models effectively deepens the model’s depth, potentially leading to gradually declining performance. These issues are worthy of in-depth consideration and exploration.

### 2.5. Road Feature Extraction Based on Multiple Tasks

**Figure 7:**
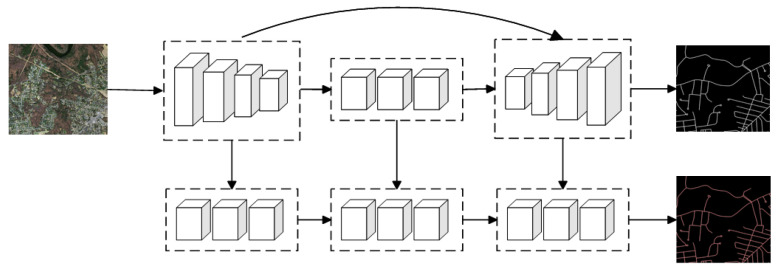
Network Models Based on Multiple tasks.

The focus of most current road extraction tasks is primarily on extracting road surfaces. However, roads encompass various elements, including road centerlines, road edges, road nodes, and more, all of which are equally important. Consequently, the challenge of achieving multi-task road extraction persists. Many researchers are exploring network models for accomplishing multi-task road extraction in remote sensing images, surpassing the scope of surface extraction alone (Figure 7).

In the road surface and centerline extraction tasks, the D-LinkNet model was employed [126]. Initially, the imagery was coarsely segmented for road extraction. Subsequently, the boosting segmentation network (BSNet) based on the ResNet-34 network architecture was used to enhance the connectivity and accuracy of the coarse segmentation results. Road intersections simultaneously generated starting points by employing multi-start point tracking. Finally, an iterative search strategy embedded with convolutional neural networks (CNNs) was used to track a continuous and complete road network. Refined extraction of road surfaces and centerlines was achieved by integrating segmentation, tracking results, semantic information, and topological data. A dual-task end-to-end convolutional neural network (MRENet) [127] with a dual-branch structure was developed. These two branches facilitated feature sharing, with the main branch responsible for road surface extraction, and the other branch utilizing features extracted from the main branch as conditions for centerline extraction. This information exchange and parameter sharing approach helped mitigate potential issues arising from insufficient centerline samples. To address the problem of poor connectivity in road extraction often caused by complex backgrounds, Lu et al. [128] identified interconnections between different extraction tasks. For example, the road surface segmentation results influenced the final position of centerlines and edges, and the integrity of road edges was closely related to road surface connectivity. Therefore, they proposed a cascaded multi-task (CasMT) road extraction framework to simultaneously extract road surfaces, centerlines, and edges. This framework fully leveraged the interrelationships between these tasks, promoting interconnectivity within the road network.

To improve the connectivity of road surfaces, additional information about roads, such as road nodes and intersections, is also extracted by many scholars in multi-task extraction. D. Chen et al. [129], while using network models to extract road surfaces, also extract information about road nodes. This node information provides supervision for road surfaces, contributing to their continuous improvement in connectivity. X. Chen et al. [130] constructed a node inference branch within the network, modeling road nodes together with road surfaces, thereby enhancing the topological structure of roads and reducing surface fragmentation. Roads and intersections are two crucial elements in road network generation. Li et al. [102] using trajectory data and remote sensing images, and not only extracted road surfaces but also recovered intersection information from road area features, simultaneously performing road surface and intersection extraction tasks. Additionally, some researchers apply multi-tasking to segmentation and change detection. M. Zhou et al. [100] proposed a neural network with dual-task road change detection, called dual-task dominant Transformer-based neural network (DT-RoadCDNet). This network takes input from two-phase remote sensing images and can perform both segmentation and change identification tasks, resulting in two road surface segmentation images before and after changes and one road change image.

Roads are not only composed of road surfaces but also include elements such as road centerlines, road edges, and road nodes. The emergence of multi-task road extraction has the potential to enhance road information, facilitating better road pipeline planning. However, in current road extraction tasks, research focused on road centerlines as the primary extraction task is relatively scarce, with most relying on labeled data provided by OpenStreetMap (OSM). Road centerlines are not only vital components of roads but can also serve as weak labels for subsequent tasks based on weak supervision learning. Additionally, road edges and road nodes are equally crucial. Edges determine the integrity and continuity of road surfaces, while linear elements consist of nodes. Nodes can be used as additional information for predicting and inferring road surface breakpoints and completing linear elements, thus improving road connectivity. They can also serve as road backbones, facilitating subsequent road vectorization processing. Road networks evolve and change each year, and electronic maps require timely updates of road networks. Traditional methods often require substantial human and material resources for field surveys. Road change detection tasks rely on neural networks and remote sensing images, automating the extraction of road changes from images, reducing the need for manual intervention. However, due to limitations in data sources and labels, change detection tasks still face issues of missed detections and false alarms, necessitating further improvement in data source quality, label quality, and network model quality.

### 2.6. Road Feature Extraction Based on Network Optimization

The various strategies employed by research scholars in optimizing the training of network models are research hotspots, and the primary focus is loss functions. Loss functions play an indispensable role in the training of network models, as they measure the difference between the model’s predictions and the ground truth. Model performance is typically evaluated by calculating the loss value, where lower loss signifies better model performance, indicating that the model’s predictions are closer to the ground truth.

We find that the dice coefficient loss, binary cross entropy loss, and cross entropy loss are the most commonly used loss functions. Since road extraction tasks are typically binary semantic segmentation tasks, binary cross entropy loss is more common than cross entropy loss. Additionally, in model training, the dice coefficient loss is used to measure the similarity between predicted results and labels, while binary cross entropy loss is employed to assess the distance between predicted results and actual labels. For instance, Lin et al. [72] introduced both of these loss functions into their proposed SE-DeepLab network and compared their effectiveness in model training. They found that the dice loss was better suited for their model, significantly enhancing its performance during training and prediction. Similarly, Lan et al. [53] also argued that the dice coefficient loss is more suitable for road segmentation tasks because it conducts global assessment, whereas binary cross entropy loss is pixel-wise. When extreme imbalance exists between foreground and background, binary cross entropy loss may not effectively address this issue. However, the dice coefficient loss is sensitive to noise and may overlook boundary information, leading to poorer road edge segmentation. To address this concern, Zao and Shi [131] proposed an edge-focused loss, which guides the network to pay more attention to road edge regions. Additionally, they introduced an enhancement factor that assigns higher loss contributions to pixels closer to the edges, thereby improving road boundary segmentation.

Different types of loss functions are combined, which is a training strategy used by the D-LinkNet. The loss functions were integrated by using various combinations of strategies [58,79,132] to fully exploit their respective advantages in road extraction. For example, Abdollahi et al. [133] introduced the VNet network model for road extraction and proposed a new dual-loss function called cross entropy and dice loss (CEDL). This loss function combines cross entropy (CE) and dice loss (DL) because cross entropy considers local information while dice loss focuses more on global information. Introducing the CEDL loss function into VNet can reduce the impact of class imbalance issues, thus improving road extraction results. Since high-resolution remote sensing images typically include complex backgrounds such as occlusion, shadows, and similar textures in the surrounding terrain, many roads are difficult to identify successfully, leading to a relatively high rate of omissions. To address this challenge, Lu et al. [128] introduced the hard example mining (HEM) loss function. This loss function, by jointly using dice and binary cross entropy loss functions, pays more attention to hard samples, enhancing road recognition and further improving road completeness.

To address the issue of sample imbalance, the focal loss function has been employed by some researchers [28,89,134]. Additionally Wei and Zhang [57] combined focal loss with the dice function. The focal loss function [135] differs from traditional cross entropy functions by focusing on resolving sample imbalances and confounding pixel categories. Abdollahi et al. [136] introduced a loss function called median frequency balancing focal loss weighted (MFB_FL) based on the focal loss function to deal with highly imbalanced datasets, where positive samples are scarce. The introduction of MFB_FL eases the burden on simple samples, allowing more time to be spent learning difficult samples, thereby improving road extraction and road vectorization results. The issue has also been addressed by some researchers through modifications to the loss function. Yang and Wang [109] added a spatial penalty term to the loss function to address the typical class imbalance issue in road extraction. Additionally, the softmax cross entropy loss (SCE), Jaccard, and Lovasz softmax (LZS) loss functions have been applied in binary road extraction tasks. J. Zhang et al. [61] combined Jaccard and cross entropy losses in the training of the SDG-LinkNet model to avoid the problem of single cross entropy easily falling into local optima. Furthermore, Sushma et al. [137] simultaneously used LZS and boundary loss functions during model training, with results showing their superiority over the mean squared error (MSE) loss.

With relatively limited research on loss functions in road extraction tasks, an attention loss function called GapLoss was proposed by Yuan and Xu [138]. This function can be combined with any segmentation network. Firstly, a binary prediction mask is obtained using a deep learning network. Secondly, a vector skeleton is extracted from the prediction mask. Thirdly, for each pixel, eight adjacent pixels with the same value are calculated, and if the value is 1, the pixel is identified as an endpoint. Fourthly, based on the number of endpoints within a buffer range, the corresponding weight is assigned to each pixel in the predicted image. Finally, the weighted average of the cross entropy of all pixels in the batch is used as the final loss function value. GapLoss was introduced into four relatively basic network models (PSPNet, U-Net++, SegNet, and MUNet), and the training results outperformed the use of the three loss functions: dice, binary cross entropy, and focal. This suggests that GapLoss not only improves the connectivity of predicted roads but also enhances the accuracy of road predictions. Xu et al. [139], based on the D-LinkNet, compared twelve well-known loss functions, categorizing them into region-based (such as dice, Jaccard, and focal), distribution-based (such as binary cross entropy), and composite-based (such as a combination of dice and binary cross entropy). They found that different loss functions performed significantly differently under different models. Region-based loss functions generally outperformed distribution-based ones, while the performances of region-based and composite-based loss functions were comparable. This indicates that the choice of the most suitable loss function should be based on the model’s design.

In addition to the utilization of loss functions for optimizing model training, the traditional batch normalization (BN) layer has been replaced with filter response normalization (FRN) in the upsampling layer by some researchers [27,140]. With the introduction of this layer, the model decreases its dependence on random batches, thereby benefiting model optimization and enhancing training efficiency.

This section primarily introduces the fundamentals of network optimization in road extraction tasks, with an emphasis on the utilization of loss functions. Additionally, it briefly mentions adjustments made between different layers of the model to enhance the model’s training capabilities. Concerning the application of loss functions, binary cross entropy, dice loss, and their combinations represent the most commonly employed loss functions in model training. However, due to variations inherent in different models, the performance of various loss functions may exhibit differences. Furthermore, it is worth noting that there is relatively limited in-depth research on loss functions in the road extraction field. Although dice loss and binary cross-entropy–dice combinations are presently regarded as more suitable loss functions, the question of whether these loss functions can consistently perform well in new models that are deeper, wider, and larger warrants consideration. Therefore, one of the future research directions involves the design of loss functions with strong generalization capabilities aimed at improving performance on diverse models.

## 3. Road Feature Extraction Based on Semi-Supervised (Weak) Deep Learning Network Models

Semi-supervised learning falls within the domain of weakly supervised learning, combining elements of both unsupervised and supervised learning. It consists of a supervised learning part and an unsupervised learning part. Zhou [141] subdivided weakly supervised learning into three categories: (1) incomplete supervision refers to the situation where only a portion of the training data are labeled, and the rest are unlabeled. (2) Inexact supervision refers to the provision of coarse-grained label information in the training data, which is more common in tasks such as object detection and instance segmentation but less prevalent in road extraction tasks, where road extraction is typically a binary semantic segmentation problem. (3) Inaccurate supervision means that the labels in the training data may contain errors or inaccuracies, which are inevitable in road datasets because road labeling typically involves manual annotation. The author proposes corresponding solutions for these three types of supervision. For incomplete supervision problems, active learning or semi-supervised learning methods are used. Additionally, multi-instance learning can be applied to address inexact supervision problems. For inaccurate supervision problems, learning with label noise strategies is employed, introducing noise to the labels for model training. In summary, both semi-supervised learning and weakly supervised learning rely on a small amount of labeled data and a large amount of unlabeled data for training models and improving performance. In the field of road extraction, researchers have used various methods to address the issue of limited labeled data. This section will explore this issue from the perspectives of weakly supervised learning and semi-supervised learning.

### 3.1. Road Feature Extraction Based on Weakly Supervised Learning

In weakly supervised road extraction tasks, the challenge of acquiring pixel-level labeled data at a high cost and difficulty is encountered by researchers. Therefore, the exploration of alternatives such as weak label data, such as point or line annotations, has become a focus. These data are comparatively easier to obtain and more abundant than pixel-level labels, making them the preferred choice for researchers. For instance, a method known as “deep windows” [142] effectively utilizes point annotation data in road centerline extraction tasks. A block-based road center point estimation model was initially designed, inspired by the stacked hourglass networks applied in the field of human pose estimation [143]. This model was then trained using point annotations (indicating the center points of roads in training blocks) to predict road center points within local blocks. Subsequently, the direction of the road was estimated using the Fourier spectrum analysis algorithm. Guided by the CNN model, road center points within blocks were iteratively tracked and connected along the road’s direction, completing the road centerline extraction. Building upon this method, Lian and Huang [144] further developed a point-based weakly supervised road segmentation method for road surface extraction. Point annotation data were initially utilized to detect road seed points and background points in remote sensing images. These points were then used to train a support vector machine classifier (SVC) for classifying each pixel in the image as road or non-road. Simultaneously, a multi-scale and multi-direction Gabor filter was introduced to estimate the road potential of each pixel based on the preliminary classification results, taking into consideration the local geometric and directional features of the road. Finally, an active contour model algorithm based on local binary fitting energy (LBF-Snake) was introduced to extract road contours from non-uniform road potential maps and optimize road regions through simple post-processing.

The weakly supervised road surface extraction method “ScRoadExtractor” was proposed [145]. This method utilizes road centerlines as line drawing label data and combines remote sensing images with a road label propagation algorithm to generate pseudo-labels. Holistically nested edge detection (HED) was employed for edge detection within the imagery boundary. Additionally, a network model with a dual-semantic branch (DBNet) was designed for training. The model’s primary branch is based on an encoder–decoder structure, with ResNet-34 serving as the encoder backbone. The intermediate layer incorporates atrous spatial pyramid pooling (ASPP). The decoder includes road surface segmentation and road boundary detection branches, which utilize segmentation and boundary loss functions to assess the similarity between the segmentation results and pseudo-labels and the edge segmentation results and edge detection. This enables the network to iteratively optimize and improve road extraction. M. Zhou et al. [146] observed that in the presence of background occlusion and spectral confusion in remote sensing images, road edges tend to appear blurry. Using single-pixel-width line drawing labels alone to approximate the position of road centerlines does not offer sufficient supervision for road boundary learning. Consequently, this results in decreased accuracy in road surface segmentation when employing line drawing supervision methods. They also considered the label propagation algorithm to be overly complex and, as a result, opted not to use it. Instead, they introduced a weakly supervised road segmentation network, SOC-RoadNet, based on structural and directional consistency. SOC-RoadNet utilizes line drawing labels as weak supervision for road surface extraction from remote sensing images. SOC-RoadNet features a dual-branch architecture, encompassing a road segmentation branch and a road direction prediction branch. The road segmentation branch directly learns road surface features from the line drawing labels, while the direction prediction branch predicts continuous road directions to enhance road connectivity. Rather than regularizing road boundaries using unreliable edge maps, SOC-RoadNet improves the accuracy of road boundaries by introducing a structural consistency loss function. These methods illustrate how to judiciously leverage point and line annotations to enhance road extraction performance and accuracy within a weakly supervised learning framework.

### 3.2. Road Feature Extraction Based on Semi-Supervised Learning

When applying semi-supervised learning to road extraction tasks, three main aspects are typically addressed. The first involves consistency regularization, often entailing two branches, each dealing with samples subject to different perturbations. Through loss functions, the predictions of these two branches are encouraged to remain consistent. This means that some form of perturbation (e.g., flipping, rotating, cropping, and mirroring) is applied to unlabeled sample data, and the model’s predictions should exhibit minimal changes. The second aspect pertains to adversarial training, wherein adversarial strategies are applied to unlabeled data to align the outputs of unlabeled data as closely as possible with the distribution of real data. Finally, pseudo-labeling is the third aspect, involving an initial model training using labeled data. Subsequently, the trained model is utilized to make predictions for unlabeled data, high-confidence samples (above a pre-defined threshold) are selected, and their predicted results are used as pseudo-labels. These pseudo-labeled data are integrated into the labeled dataset, and the model undergoes further training on this expanded labeled dataset through an iterative process aimed at ongoing model optimization. In general, these methods are aimed at addressing challenges such as limited label availability and high annotation costs.

(1) Based on the consistency regularization

When applying semi-supervised learning to road extraction tasks, the three approaches mentioned above have been utilized by researchers. For instance, the introduction of the idea of consistency regularization into road extraction was presented [147]. A semi-supervised semantic segmentation method for fine-grained road scene understanding was designed. Four perturbation strategies were employed, encompassing random grayscale, random blur, random color jitter (brightness, contrast, saturation, etc.), and random Gaussian noise. A dual-branch structure was implemented, with one branch perturbing unlabeled data and the other branch preserving the original image. The combination of labeled and unlabeled samples in a U-Net model, with a balanced strategy of supervised and unsupervised losses, enabled the efficient extraction of road scene information, including vehicles, road lines, crosswalks, ground markings, and lane widths. This approach not only improved the classification accuracy of semantic segmentation networks but also mitigated the negative impact of limited labeled data on network performance. In another study [148], which focused on consistency regularization in semi-supervised learning, perturbation schemes were reviewed, and prominent data-level perturbation schemes, CutMix and ClassMix (a development from CutMix), as well as model-level perturbation representatives, mean teacher (MT) and cross pseudo-supervision (CPS), were identified. Inspired by these four perturbation methods, an end-to-end semi-supervised semantic segmentation framework named “ClassHyPer” was proposed. This framework is based on the ClassMix structure and simultaneously incorporates MT and CPS perturbations to form a mixed perturbation strategy. The images subjected to these mixed perturbations were then processed through a classic FCN with VGG16 as the backbone structure. By employing various loss functions to calculate sample correlations, ClassHyper exhibited strong performance on five different urban and road datasets, demonstrating its potential in enhancing model performance when confronted with limited labeled data.

(2) Based on the consistency regularization and pseudo-labels

The concept of consistency regularization and pseudo-labeling was introduced into semi-supervised road extraction tasks by You et al. [149], who proposed a novel semi-supervised remote sensing road extraction method called “FMWDCT”. This method comprises two key components: dual-network cross training (DCT) and foreground pasting (FP). The objective of dual-network cross training is to address common challenges in remote sensing image segmentation tasks, such as limited training data and high annotation costs. Foreground pasting involves the integration of foreground pixels from labeled images into unlabeled images, generating mixed input images. This strategy aims to tackle the issue of imbalanced positive and negative training samples in road extraction tasks. In FMWDCT, each network includes both an initial network and an enhancement network. Mixed pseudo-labels are generated by combining high-confidence predictions from the enhancement network and labeled masks. Subsequently, these mixed pseudo-labels are employed to guide cross training in another adversarial base network and to facilitate smoothing updates in the corresponding enhancement network. This approach contributes to the enhancement of road extraction in situations involving limited labeled data while harnessing the potential of unlabeled data and pseudo-labeling.

(3) Based on adversarial training and pseudo-labels

The semi-supervised road extraction problem was addressed [150] through the utilization of adversarial training and pseudo-labeling. They introduced an innovative semi-supervised road extraction network known as “SemiRoadExNet”, which is designed based on generative adversarial networks (GANs) and comprises a generator and two discriminators. The generator follows an encoder–decoder structure, utilizing ResNet-34 as the encoder backbone, and introduces channel attention and spatial attention in a serial strategy. Additionally, multiple dilated convolutions with skip connections are incorporated in the middle layers. Two discriminators, based on the U-Net architecture, are employed for different tasks. The working principle of SemiRoadExNet is as follows: first, labeled and unlabeled images are input into the generator network for road extraction. The generator’s output includes road segmentation results and their corresponding entropy maps. The entropy map represents the confidence level for each pixel’s prediction of road or non-road. Next, two discriminators are utilized to enforce the consistency of feature distributions between the road prediction maps and entropy maps of labeled and unlabeled data. Through adversarial training, the generator is continuously regularized, exploring latent information within unlabeled data and enhancing the model’s generalization capability. This method aims to maximize the utilization of potential information in low-confidence pixels in pseudo-labels, further enhancing semi-supervised road extraction models, reducing reliance on labeled data, and improving network performance.

### 3.3. Road Feature Extraction Based on Semi-Weakly Supervised Learning

A novel approach [151] combines the strengths of semi-supervised and weakly supervised learning, resulting in a method known as semi-weakly supervised learning. In this context, adversarial training from semi-supervised learning and the utilization of weak labels (such as road centerlines) from weakly supervised learning were leveraged to propose a remote sensing image road extraction model named “SW-GAN”. SW-GAN comprises two generators and one discriminator. These generators include a fully supervised generator based on the D-LinkNet model and a weakly supervised generator based on the Res-UNet model, which incorporates learnable pyramid dilated modules into the middle and skip connection layers to expand the receptive field. The training dataset includes both fully supervised and weakly supervised datasets. During the training process, the fully supervised generator uses both the fully supervised and weakly supervised datasets, while the weakly supervised generator utilizes only the weakly supervised dataset. The output of the weakly supervised generator is employed as a feature to augment the fully supervised generator. To ensure consistency between the fully supervised and weakly supervised generators on the weakly supervised dataset, a consistency loss function is designed to encourage both generators to produce results that are as similar as possible. The discriminator employs an FCN model, aiming to distinguish whether the generated road network is a pixel-level manually annotated road network or fully supervised synthesized road network. SW-GAN effectively utilizes a limited amount of fully supervised data and a substantial amount of weakly supervised data for road network extraction in remote sensing images, combining the advantages of semi-supervised and weakly supervised learning and achieving outstanding road extraction results.

## 4. Discussions

This paper starts from the perspective of supervised learning in deep learning, emphasizing the technical intricacies involved in road extraction from remote sensing images, and categorizes supervised learning into four methods based on the use of pixel-level label data. The advantages and disadvantages of the four learning methods are listed in Table 4.

For a more comprehensive evaluation of model performances, we primarily assess the accuracy of the models based on five key metrics, namely intersection over union (IoU), overall accuracy (OA), Precision, Recall, and F1. IoU indicates the overlap between the predicted and ground truth road areas in road extraction tasks. OA denotes the accuracy, signifying the ratio of correctly predicted pixels to the total pixels. Precision reflects the proportion of accurately predicted road pixels by the model, while Recall measures the number of roads identified by the model. F1 is the harmonic mean of Precision and Recall. Simultaneously, we have outlined the performance of several models on the road dataset of Massachusetts, as depicted in Table 5.

LDANet [97] demonstrates exceptional performance in terms of Recall, Precision, and F1-Score, showcasing its ability to accurately identify road pixels while effectively reducing false positives. Furthermore, LDANet boasts an impressively low parameter count of only 0.2M, positioning itself as an outstanding lightweight model, thereby highlighting a promising direction for future research and adoption. Seg-Road-I, DU-Net, CM-FCN, and others exhibit commendable performance across multiple metrics, showcasing elevated levels of Recall, Precision, and F1-Score. Similar to LDANet, they serve as representatives of high-performance models in this domain.

ConSwin, DCANet, and DiResNet all have overall accuracy (OA) exceeding 98%. This high OA indicates that these models exhibit a very high level of accuracy in correctly classifying road and non-road pixels within the dataset they were evaluated on.

Prop-GAN, DCANet, and Seg-Road-I exhibit high mIoU, with Prop-GAN achieving the highest mIoU among these models. This signifies their robustness and precision in road extraction tasks, indicating their capability to accurately identify and extract road information.

In conclusion, we have provided a more detailed summary of the limitations and challenges associated with current models in the context of road extraction. The following points encapsulate our findings:

(1) Model Complexity vs. Inference Speed

Complex models generally confer superior accuracy, however, at the potential expense of increased computational overhead and a higher number of parameters during the inference phase. Looking forward, achieving a nuanced equilibrium between model complexity and predictive speed is imperative, particularly in the context of real-time applications for road extraction.

(2) Generalization vs. Specialization

When confronted with unfamiliar road data, models demonstrating excessive specialization may encounter challenges, while those characterized by an overly generalized nature may fail to comprehensively capture the nuanced complexities within specific road domains. Achieving a judicious balance is crucial for optimizing performance across diverse road scenarios.

(3) Interpretability vs. Model Performance

Simplified models are often prized for their interpretability, yet they may fall short of matching the performance of their more intricate counterparts. While road extraction may superficially appear as a straightforward binary classification task, certain deep neural networks—especially sophisticated architectures like the Transformer—are frequently characterized as “black-box” models. This characterization poses challenges in deciphering their decision making processes and assessing their suitability for deployment in binary classification tasks. Furthermore, we underscore the notion that employing overly complex models for ostensibly simple tasks might be construed as an instance of “overengineering”. Therefore, meticulous consideration is warranted in the selection of models, navigating the delicate balance between interpretability and performance.

## 5. Prospects

Despite significant progress in the field of road extraction from remote sensing images in recent years, there are still some issues that require further research and development, summarized as follows:

(1) Obtaining High-Quality Labeled Sample Data

This can be addressed by employing semi-supervised and weakly supervised learning methods, combining limited labeled sample data with a large amount of unlabeled data. Although these methods may not achieve the same level of accuracy in road extraction as full supervision, they provide new approaches to addressing this challenge. Furthermore, we have observed that there is a relatively limited availability of open road datasets in complex mountainous terrains when organizing the dataset. Therefore, there is a need to further expand data resources in this regard.

(2) Differences in Spectral Information Due to Factors Such as Sensors and Solar Angles

Additionally, when dealing with challenges like road occlusion and complex background information, relatively simple neural networks can be employed to separate road and non-road areas in advance, thereby enhancing the robustness of the model in subsequent recognition tasks. However, it is worth noting that research in areas such as image denoising and super-high-resolution reconstruction remains relatively limited in the field of data enhancement.

(3) Utilizing Multi-Modal Data

Currently, the application of multi-modal data in road extraction research is relatively limited. Multi-spectral (hyperspectral) data provide us with rich spectral information, while SAR data compensate for the limitations of optical images when dealing with issues like vegetation occlusion. However, LiDAR data are distinctive, typically in the form of three-dimensional point cloud data, and there are significant differences in spatial representation compared to two-dimensional road data. Therefore, further research is needed in the area of data fusion. Scholars in this field have conducted relatively limited research, leaving room for further exploration in the future. With the continuous expansion of crowdsourced data and the advantages of GNSS and other trajectory data, which do not contain additional environmental information and have minimal interference, they have played a significant role when combined with optical images. This combination provides us with complementary information and effectively mitigates issues such as the loss of road intersection information and incomplete connections. In the future, crowdsourced datasets from platforms like Google, Amap, Didi, Baidu, and others will further support and assist road extraction.

(4) Optimization of Fully Supervised Learning Models

From generative adversarial networks (GANs) to conditional generative adversarial networks (CGANs), and from unsupervised learning to supervised learning, these advancements all emphasize the advantages of supervised learning in road feature extraction to achieve more ideal road extraction results. Models based on the encoder–decoder structure are still a popular research direction in the current deep learning field. Introducing attention mechanism modules in different structures, achieving multi-scale feature fusion, considering the introduction of Transformer, GCNs, and deep convolutional separation structures, and even introducing corresponding loss functions based on the model’s characteristics during the training process all contribute to improving the model’s road feature extraction performance in images. As models move towards greater depth and width, an increase in model size may lead to an excess of parameters, thereby raising training costs. Therefore, seeking lighter, more efficient, and more highly generalizable models becomes an important direction for future research.

(5) Optimization of Semi-Supervised (Weak) Learning Models

With the emergence of semi-supervised (weak) learning, we have successfully overcome the challenges of high costs and the difficulty of obtaining labels by using a small amount of labeled data and a large amount of weakly labeled annotation data. We have employed various methods and strategies for model training, achieving training results approximating those of fully supervised learning. However, despite the significant progress made in semi-supervised and weakly supervised learning, there is still a substantial gap in accuracy when it comes to road extraction compared to fully supervised learning. Additionally, there is relatively limited research on models based on semi-weakly supervised learning. Therefore, future research directions should explore how to fully integrate the respective strengths of semi-supervised and weakly supervised learning to compensate for their shortcomings and build more powerful semi-weakly supervised models.

(6) Road Extraction Post-Processing

Road segmentation is not the end of road extraction. After road segmentation, there is still significant room for the post-processing of road extraction. This is because the quality of the model’s extraction cannot be solely measured by high or low accuracy. Further observation is required to assess whether the connectivity of roads in the image is intact or if there are issues like fragmentation. Relevant post-processing methods can be used to repair damaged roads and improve the connectivity of poorly connected intersections. Additionally, attention should be given to specific tasks such as vectorization of roads, estimation of road areas, and registration of road features with aerial imagery. These tasks are of great significance to fields such as geographic information systems (GISs), urban road networks, and electronic map updates. Conditional generative adversarial networks (CGANs) can be applied not only to road extraction tasks but also provide new avenues for road extraction post-processing. By utilizing the differences between the generator and discriminator backbone models and additional conditions like adding noise and artifacts, they offer extensive opportunities for the future development of post-processing in this field.

## Figures and Tables

**Table 1:** Attention Mechanisms and Methods.

Model/Method	Attention Mechanism	Highlight(s)/Strength(s)
ConSwin-Net [69]	Multi-Head Self-Attention	Introduction of dual Swin Transformers and a residual block within the U-Net network structure, creating the ConSwin-Net, which mitigates CNN limitations in extracting global contextual features, thereby enhancing the model’s perception of road texture details and global information
Seg-Road [70]	Self-Attention	Incorporation of a Transformer structure into the encoder combined with a convolutional neural network (CNN) decoder leads to improved connectivity in road segmentation and enhanced prediction result robustness
FSNet [71]	Self-Attention	Integration of the self-attention feature transfer module (SAFM) into the hidden layers of convolutional neural networks establishes relationships between each hidden layer and its contextual hidden layers. This facilitates the transfer of hidden layer feature information to the original feature map, resulting in improved road extraction performance
Nested SE-DeepLab network [72]	Channel Attention (SE)	Introduction of SE module into the encoder and decoder effectively merges and retains both shallow and deep information, addressing model imbalance issues in narrow road extraction
DSDNet [29]	Channel Attention (SE)	Integration of the SE module into the encoder of the D-LinkNet network assists ResNet in feature extraction for mountain roads
TSE-LinkNet [54]	Channel Attention (SE)	Combining the SE module with the ASPP module during downsampling enhances topological relationships between adjacent road pixels in images
BMDANet [75]	Modified Efficient Channel Attention (MECA)	Utilization of the improved MECA module enhances the continuity of road features based on the characteristics of RSI roads
MSACon [76]	Spatial Attention (SAM)	Construction of an MSACon dual-encoder network with a spatial attention-based fusion (SAF) mechanism improves road extraction by utilizing contextual relationships between roads and buildings
RALC-Net [1]	Spatial Attention (SAM)	Development of a dual-encoder RALC-Net network with a residual attention (RA) module integrates spatial contextual information to emphasize local semantics, aiding in the extraction of local road features
GCB-Net [28], CDG [77], CADUNet [78]	Global Attention (GA)	Focusing on highlighting high-level road features to improve segmentation results
CADUNet [78]	Core Attention (CA)	Ensuring the maximum transmission of road information between dense blocks and coordinating multi-scale road information acquisition through the global attention module
SANet [55]	Strip Attention (SAM)	Facilitating the fusion of lower-level and higher-level road features
FE-LinkNet [59]	Criss-Cross Attention (CCA)	Enhancing pixel-level representation capabilities by capturing long-range contextual information in horizontal and vertical directions
SegRExt-F [67]	Convolutional Block Attention Module (CBAM)	Improving network focus on images through concatenation of channel and spatial attention using CBAM
DU-Net [74]	Pro Convolutional Block Attention Module (ProCBAM)	Enhancing the integration of road information through ProCBAM with added SE module
SDG-LinkNet [61]	Position Attention Module (PAM) with D-Blockplus	Introducing the position attention module (PAM) and global information recovery module (GIRM) in parallel for global information acquisition
Meca-Net [66]	Long-Range Context-Aware Module (LCAM)	Designed to alleviate road occlusion issues by acquiring long-range context information through channel and spatial attention
GAN [79], MAU-Net [80], GAMSNet [81], CM-FCN [82]	Parallel Channel and Spatial Attention	Enhancing road information extraction and segmentation performance through the integration of parallel channel and spatial attention
MAU-Net [80]	Feature Fusion based on Attention Mechanism (FFBAM)	Introduced a feature fusion mechanism (FFBAM) for better fitting multi-scale road information
BMDANet [75]	Block Multi-Dimensional Attention (BMDA) Module	Introduced BMDA for feature extraction in blocks, integrating them through channel and spatial attention
CMAFE [83]	Cascaded Multi-Scale Attention Feature Enhancement (CMAFE)	Coarse feature extraction with dilated convolution pooling, followed by boundary enhancement in the lightweight U-Net network
Rse-Net [84]	Multi-Scale Convolutional Attention Module (CSAM)	Introduced Rse-Net with multi-scale convolutional attention module, focusing on boundary information and expanding the receptive field for more semantic information

**Table 2:** Multi-Scale Feature Fusion Module and Methods.

Model/Method	Multi-Scale Feature Fusion Module	Highlight(s)/Strength(s)
Geographic Feature-Enhanced Network [92]	Joint Shared Learning and Feature Fusion	Enhancing road extraction connectivity through joint learning of pixel-level, edge-level, and region-level road features, followed by feature fusion
DA-CapsUNet [89]	Multi-Scale Context Augmentation (CTA)	Enlarging the receptive field and integration of context information from different scales
BT-RoadNet [90]	Coarse Map Predicting Module (CMPM) and Spatial Context Module	Serially connected spatial context module effectively captures elongated road shapes
Deep FR TransNet [91]	Feature Review (FR)	Evaluating road features of varying scales, with an emphasis on contour characteristics, to improve road profile information
DCANet [93]	Discriminative Context-Aware Feature (DCF) Module	Aligning feature maps across scales to extract high-frequency information, with a refine decoder (RD) for spatial information retention and feature representation
AF-Net [94]	All-Scale Feature Fusion (AF) Module	Recursive integration of features from two pathways, leveraging scale features with varying spatial and semantic information, to provide accurate spatial and semantic information for road extraction
NFSNet [71]	Global Feature Refinement (GFR) Module	Improved semantic information of feature maps for more detailed segmentation outputs
ConSwin-Net [69]	Feature-Enhanced Connection (FC) and Shape-Augmented Connection (SC)	Enhanced and separate transmission of structural and textural features to the decoder, improving overall model performance
MLEM-NET [95]	Multi-Scale Line Enhancement Module (MLEM)	Utilizing the Hough transform (HT) to enhance local and global linear features in remote sensing images
SDUNet [96]	Densely Connected Encoder Block and Spatial Intensifier (DULR) Module	Constructing spatial relationships between features at different positions and introducing skip connection layers to preserve the topological structure
Meca-Net [66]	Multi-Scale Feature Encoding Module (MFEM)	Utilizing convolution kernels of different scale sizes and aggregating multi-scale features through a parallel strategy for recognizing elongated roads
MSPFE-Net [57]	Feature Enhancement Module (FEM) with Stripe Pooling	Extracting and merging features from various levels to accomplish multi-scale feature fusion
LDANet [97]	Feature Expansion Module and Deep Feature Association Module	Expanding and merging features to address challenges posed by narrow and complex rural roads, improving feature associations, and promoting multi-feature fusion
MTMF [98]	Canny Operator and HRNet	Improving road image segmentation through the fusion of edge information and image features

**Table 3:** Multi-modal Fusion Module and Methods.

Model/Method	Module Name	Fusion Data Sources	Highlight(s)/Strength(s)
DeepDualMapper [101]	Gated Fusion Module (GFM)	GPS trajectory data and remote sensing imagery	GFM was designed to control and integrate information from both modalities in a complementary perception manner
MTMSAF [102]	Adaptive Fusion Module (AFM)	GPS trajectory data and remote sensing imagery	AFM was utilized to integrate road features from trajectory data and remote sensing imagery
CMMPNet [103]	Dual Enhancement Module (DEM)	Cross-source fusion of images and trajectory data	DEM was introduced to enhance and complement features from both images and trajectory data bidirectionally, applicable for LiDAR and remote sensing imagery data
MSFANet [104]	Cross-source Feature Fusion Module (CFFM)	Traditional remote sensing imagery and hyperspectral imagery	Hyperspectral and remote sensing imagery are combined to alleviate discontinuous outputs and using CFFM to correct and fuse spectral features at different scales, reducing noise and redundancy

**Table 4:** Comparison of 4 learning methods.

Learning Type	Labeled Data Usage	Extraction Accuracy	Generalization Ability	Prospects for Future Research	Disadvantages
Fully Supervised Learning	Large amount of high-quality labeled data	High accuracy	Relatively poor	Excellent results with sufficient labeled data, limited generalization	Requires substantial human effort and cost to label data. It may overfit to labeled data and lack adaptability to unseen scenarios
Semi-Supervised Learning	Small amount of labeled data + unlabeled data	Lower than fully supervised	Better than fully supervised	Potential improvements through utilizing both labeled and unlabeled data	Complexity in designing algorithms that effectively leverage both labeled and unlabeled data, risk of error propagation from weak labels
Weakly Supervised Learning	Large amount of weakly labeled data	Lower than fully supervised	Strong generalization	Promising due to ease of obtaining weak labels and better generalization	Difficulty in ensuring accuracy due to the noise or ambiguity present in weak labels, potential inconsistency in labeling quality
Semi-Weakly Supervised Learning	Combination of small amount of labeled data + large amount of weakly labeled data	Moderate accuracy	Strong generalization	Opportunity to harness the benefits of both labeled and weakly labeled data	Balancing accuracy from labeled data with generalization from weak labels, potential challenges in harmonizing the different types of labeled data

**Table 5:** The Performance Comparison of Models on the Massachusetts Dataset.

Method	Recall	Precision	F1-Score	OA	IoU	mIoU	Parameters (M)
SegRExt-A [67]	68.29	76.95	-	97.53	56.82	-	-
SegRExt-F [67]	63.84	74.88	-	96.62	52.85	-	-
MSPFE-Net [57]	75.50	73.11	74.29	-	59.09	-	-
LDANet [97]	97.07	97.55	97.31	-	68.34	-	0.20
SemiRoadExNet [150]	-	-	70.23	-	54.66	-	-
Seg-Road-I [70]	92.86	87.34	90.02	-	68.38	83.89	28.67
DU-Net [74]	96.96	97.48	96.72	-	-	67.05	-
SR [31]	77.50	80.41	78.93	-	-	65.30	-
MECA-Net [66]	78.19	80.63	79.39	-	65.82	-	-
GA-Net [130]	76.89	84.10	80.33	-	67.13	-	
SDG-DenseNet [61]	77.67	81.86	79.63	-	66.47	-	265.00
SDUNet [96]	75.70	81.20	78.40	-	74.10	-	80.24
MUNet [138]	-	-	67.40	97.20	-	74.00	-
U-Net++ + Resnext [152]	95.10	94.30	94.70	-		-	
Deep residual U-Net [153]	80.00	84.00	81.00	-	72.00	-	-
CM-FCN [82]	77.87	79.45	78.65	97.98	67.55	-	56.45
CRAE-Net [83]	79.35	80.04	79.52	-	66.27	-	49.18
SGCN [154]	73.91	84.82	78.99	-	81.65	65.28	42.73
Richer U-Net [131]	-	-	-	-	58.63	-	-
GDCNet [122]	71.21	84.43	-	-	62.94	-	-
ConSwin [69]	79.17	81.11	80.13	98.15	66.84	-	-
RALC-Net [1]	-	-	74.70	-		59.61	
RoadVecNet [136]	-	-	92.51	-	86.31	-	-
MCG-UNet [119]	86.59	91.18	88.74	-	79.92	-	
AEML U-Nets [125]	76.33	81.06	78.62	-	64.77	-	-
RVgg19 [155]	91.02	84.98	87.90	-	-	-	-
CADUNet [78]	76.55	79.45	77.89	98.00	64.12	-	-
AF-Net [94]	-	-	-	-	67.25	-	-
E-UNet [118]	81.30	80.71	80.45	97.59	68.56	-	-
DCANet [93]	79.54	80.20	79.84	98.09	66.45	82.23	11.1
Deep FR TransNet [91]	78.13	83.72	-	97.48		62.86	-
Prop-GAN [7]	92.92	91.54	92.20	-		87.43	-
DGRN [56]	71.97	-	76.59	-	62.48	-	-
CNN-Based [126]	85.88	78.47	-	-	78.65	-	-
Nested SE-Deeplab [72]	-	85.80	85.70	96.70	73.87	-	-
DiResNet [124]	79.41	80.38	79.70	98.13	-	-	-
CDG [77]	71.80	81.41	76.10	-	61.90	-	-
VNet+CEDL [133]	-	-	91.18	-	83.82	-	-

## Data Availability

Data will be made available on request.
